# Revisiting the Children-of-Twins Design: Improving Existing Models for the Exploration of Intergenerational Associations

**DOI:** 10.1007/s10519-018-9912-4

**Published:** 2018-06-30

**Authors:** Tom A. McAdams, Laurie J. Hannigan, Espen Moen Eilertsen, Line C. Gjerde, Eivind Ystrom, Fruhling V. Rijsdijk

**Affiliations:** 1 Social, Genetic & Developmental Psychiatry Centre, Institute of Psychiatry, Psychology & Neuroscience, King’s College, De Crespigny Park, Box PO80, SE5 8AF London, UK; 2 Department of Mental Disorders, Norwegian Institute of Public Health, Oslo, Norway; 3 Department of Psychology, University of Oslo, Oslo, Norway; 4 School of Pharmacy, University of Oslo, Oslo, Norway

**Keywords:** Children-of-twins, Extended family design, Intergenerational transmission, Parent, Offspring, The Norwegian Mother and Child Cohort Study (MoBa), The Intergenerational Transmission of Risk (IToR) project

## Abstract

Datasets comprising twins and their children can be a useful tool for understanding the nature of intergenerational associations between parent and offspring phenotypes. In the present article we explore structural equation models previously used to analyse Children-of-Twins data, highlighting some limitations and considerations. We then present new variants of these models, showing that extending the models to include multiple offspring per parent addresses several of the limitations discussed. Accompanying the updated models, we provide power calculations and demonstrate with application to simulated data. We then apply to intergenerational analyses of height and weight, using a sub-study of the Norwegian Mother and Child Cohort (MoBa); the Intergenerational Transmission of Risk (IToR) project, wherein all kinships in the MoBa data have been identified (a children-of-twins-and-siblings study). Finally, we consider how to interpret the findings of these models and discuss future directions.

**Electronic supplementary material:**

The online version of this article (10.1007/s10519-018-9912-4) contains supplementary material, which is available to authorized users.

Studying samples of twin pairs with children can provide valuable insight into the nature of intergenerational associations. Using samples of monozygotic (MZ) and dizygotic (DZ) twins with children (or indeed any samples of differentially related siblings/cousins with offspring), it is possible to evaluate hypotheses regarding the nature of intergenerational transmission and ask whether associations between parent and child remain after accounting for their genetic relatedness.

The use of samples of twins with children to examine intergenerational associations has been discussed in depth elsewhere (D’Onofrio et al. 2003; Fischer 1971; Gottesman and Bertelsen 1989; McAdams et al. 2014; Silberg and Eaves 2004).^1^ However, briefly, the offspring of MZ twins are as related to their parent’s co-twin as they are to their own parent (they share 50% of their DNA). In contrast, the offspring of DZ twins share 25% of their genetic variance with their parent’s co-twin. By comparing MZ avuncular correlations (correlations between uncle/aunt and niece/nephew) with DZ avuncular correlations, it is possible to estimate the role of genetic factors in explaining intergenerational associations. It is also possible to estimate the extent to which parent–child associations remain after accounting for genetic transmission.

While twins with children have most often been the focus of genetically informed intergenerational studies, it is also possible to use siblings or even cousins with children, wherever samples are large enough. That is, rather than (or as well as) comparing avuncular relationships in MZ and DZ families, siblings, half-sibling and cousins can be used. Children share 25, 12.5 and 6.25% of their genetic variance with their parent’s sibling, half-sibling and cousin respectively. These differences in relatedness can be used to estimate the role of genetic factors in explaining intergenerational associations in the same way that twin data is used (e.g. Kuja-Halkola et al. 2014).

A variety of techniques can be used to analyse children-of-twin (CoT) and/or children-of-sibling (CoS) data and investigate the nature of intergenerational associations. While various multilevel regression models have been applied to such data, in keeping with behavioural genetic tradition, structural equation models (SEMs) have been most widely used, and it is these models that we focus on in this article. Biometric SEMs are advantageous in that they allow researchers to quantify the relative importance of genetic and environmental influences in explaining variance on a trait and covariance between traits. Our intention in this article is to describe the SEMs thus far applied to CoT data, discuss their limitations, and suggest extensions to the models that help to overcome these limitations. We then apply these extended models, first demonstrating their power using simulated data, and then using real data originating from the Norwegian Intergenerational Transmission of Risk (IToR) project, a sub study of the Norwegian Mother and Child Cohort Study (MoBa) (Magnus et al. 2016). Throughout, we highlight and discuss remaining limitations and issues to consider for those interested in making use of CoT models and those interested in collecting CoT and/or CoS data. We provide OpenMx scripts for all models via the Supplementary Materials section of this article.

We believe that now is a good time to revisit and explore extensions to these models for two reasons. First, intergenerational datasets now exist that are appropriate for the application of CoT models, but that are also larger and more complex than previous datasets (e.g. registry data, ITOR). Second, many on-going twin studies have participants who are in, or are entering, adulthood. When these participants begin having children, such datasets will have the potential to teach us a great deal about the nature of intergenerational transmission. It is therefore worthwhile exploring how best to model these associations, and how best to collect data for this purpose.

## Children of twins structural equation models

**Fig. 1:**
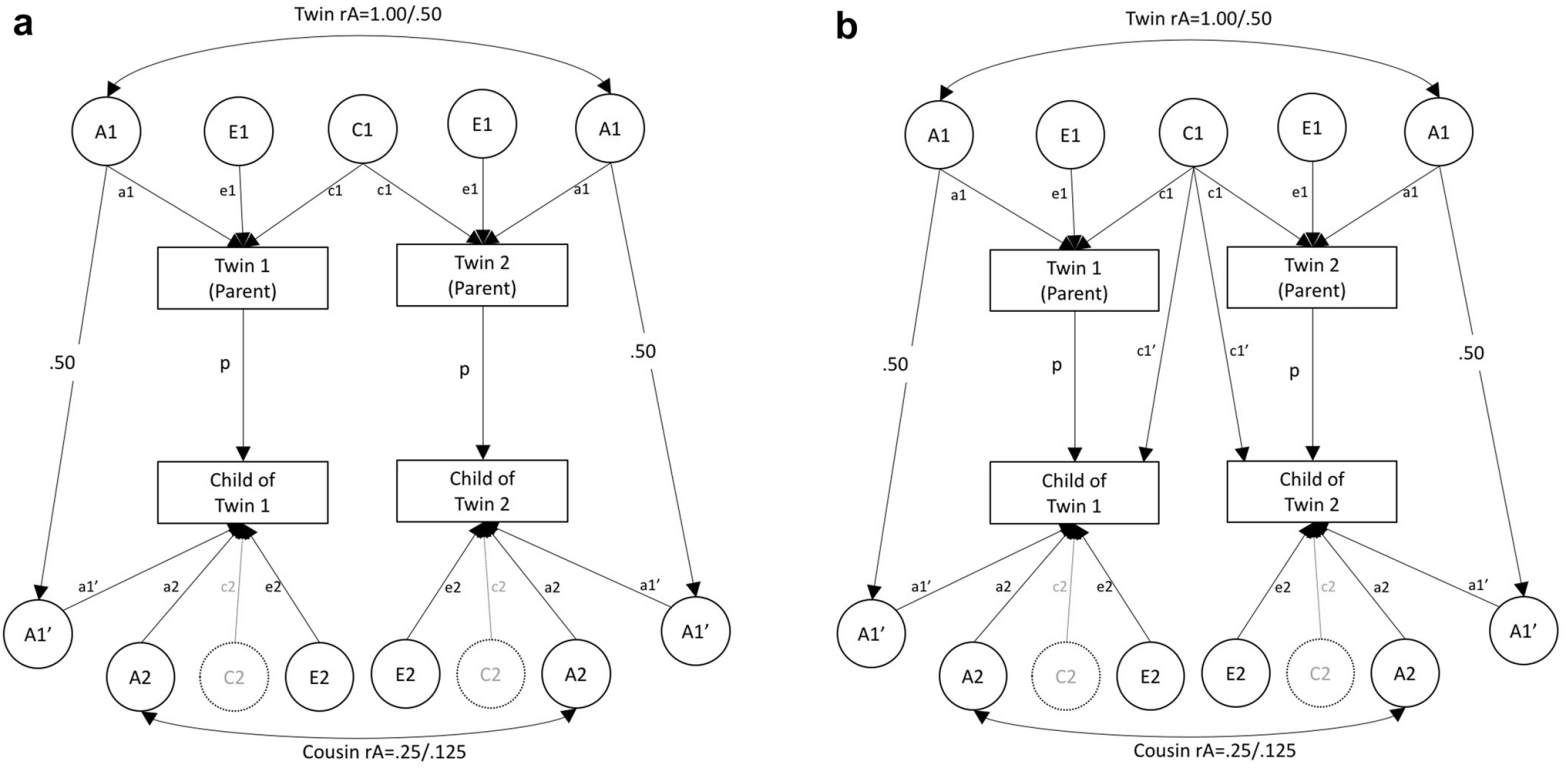
Children-of-twins structural equation models—for use with samples comprising twin pairs with a single child per twin. *Note*: A1 = additive genetic effects on parental phenotype; C1 = shared-environmental effects on parental phenotype; E1 = nonshared environmental effects on parental phenotype; A1′ = genetic effects common to parental phenotype and offspring phenotype; C1′ = extended family effects whereby the shared environment of the parents influences offspring phjenotype; A2 = familial effects specific to offspring phenotype; C2 = shared-environmental effects on offspring phenotype (not estimable using cousin data); E2 = nonshared environmental effects on offspring phenotype; p = phenotypic effect of parent on offspring; rE = within-parent correlation between E1 for parenting of child 1 and 2. Allows parenting of each child to differ (when necessary this should be allowed to vary according to offspring zygosity). NB the pathway between A1 and A1′ is fixed to 0.50 because parents and children share 50% of their genome. To avoid over complicating path diagrams, variance paths have been omitted, but for all latent factors variance = 1. For A1′ this means that residual variance (after accounting for the path between A1 and A1′) is 0.75

Several CoT SEMs have previously been published. In Fig. 1a, we present a path diagram of the model applied in several published articles to data from the Twin Offspring Study in Sweden (TOSS) (Eley et al. 2015; McAdams et al. 2015, 2017). TOSS is a study of adult twins and their adolescent children (Neiderhiser and Lichtenstein 2008). The TOSS sample comprises data collected on one child per twin, these children being cousins to one another. The model in Fig. 1a decomposes variance on the parental (twin) phenotype into additive genetic influences (A1; MZ twins share all genetic influences, DZ twins share half), common or shared environmental influences (C1; environmental influences shared between MZ and DZ twins alike), and non-shared or unique environmental influences (E1; environmental influences that make twins different to one another). Variance in the offspring phenotype is decomposed into genetic influences shared in common with the parental phenotype (A1′), genetic influences specific to the offspring phenotype (A2), and non-shared environmental influences (E2). A path also runs from parent phenotype to offspring phenotype (p). Parent–child covariance is therefore explained by a combination of p, a1′ and a1. Genetic transmission is modelled via the a1 and a1′ path, and a path fixed to 0.50. This is because parents pass on half of their DNA to their children. Where a1′ is significant, this indicates that genetic factors influencing the parental phenotype also explain variance in the offspring phenotype, and the association between parent and child phenotype is (at least partially) attributable to shared genes. Where p is significant, this indicates that the parent–child association (at least partially) persists after accounting for shared genes.

The model in Fig. 1a and other CoT/CoS models like it decompose the intergenerational association into a genetic component and a phenotypic component (Eley et al. 2015; Hannigan et al. 2016; McAdams et al. 2015, 2017; Silberg et al. 2010, 2012). In Fig. 1b we adapt this model to allow for the possibility that shared environmental influences on the parent generation may influence the offspring generation as well, via a new path, c1′, that captures the effect of the extended family environment. Parent–child covariance is thus explained by a combination of p, a1′, a1, c1′ and c1. The effects of the extended family environment have been included in suggested CoT models previously (D’Onofrio et al. 2003). Indeed, it is possible to model multiple sources of shared environment using CoT/CoS data. Intergenerational models including a variety of shared environmental factors have previously been applied to CoS data derived from the Swedish national population registries (Chang et al. 2014; Kuja-Halkola et al. 2014; Latvala et al. 2015). In the present study we include one intergenerational shared-environment path and explore the impact that significant extended family environmental effects may have on CoT models.

A key limitation of the models presented in Fig. 1a, b is that they have low power to accurately estimate offspring aetiology. This is because the offspring of twins are cousins with one another so share, on average, 25% of their genetic variance (where their parents are MZ twins), or 12.5% of their genetic variance (where their parents are DZ twins). These relatedness coefficients (0.25 and 0.125), and the difference between them, are lower than those of twins (1.00 and 0.50) and result in considerably less power to detect genetic effects unique to the offspring generation than there is for the parent generation. Similarly, the estimation of A1′ (genetic effects shared between parent and child) relies upon comparisons between MZ and DZ avuncular (aunt/uncle-to-niece/nephew) relationships, comprising relatedness coefficients of 0.50 and 0.25, so again, there is less power to detect these paths than there is to detect e.g. a genetic correlation between two phenotypes in the parent generation. Because cousins do not share a nuclear family environment it is also not possible to estimate the role of environmental effects shared by siblings in the offspring generation (C2).

## Multiple-children-of-twins structural equation models

**Fig. 2:**
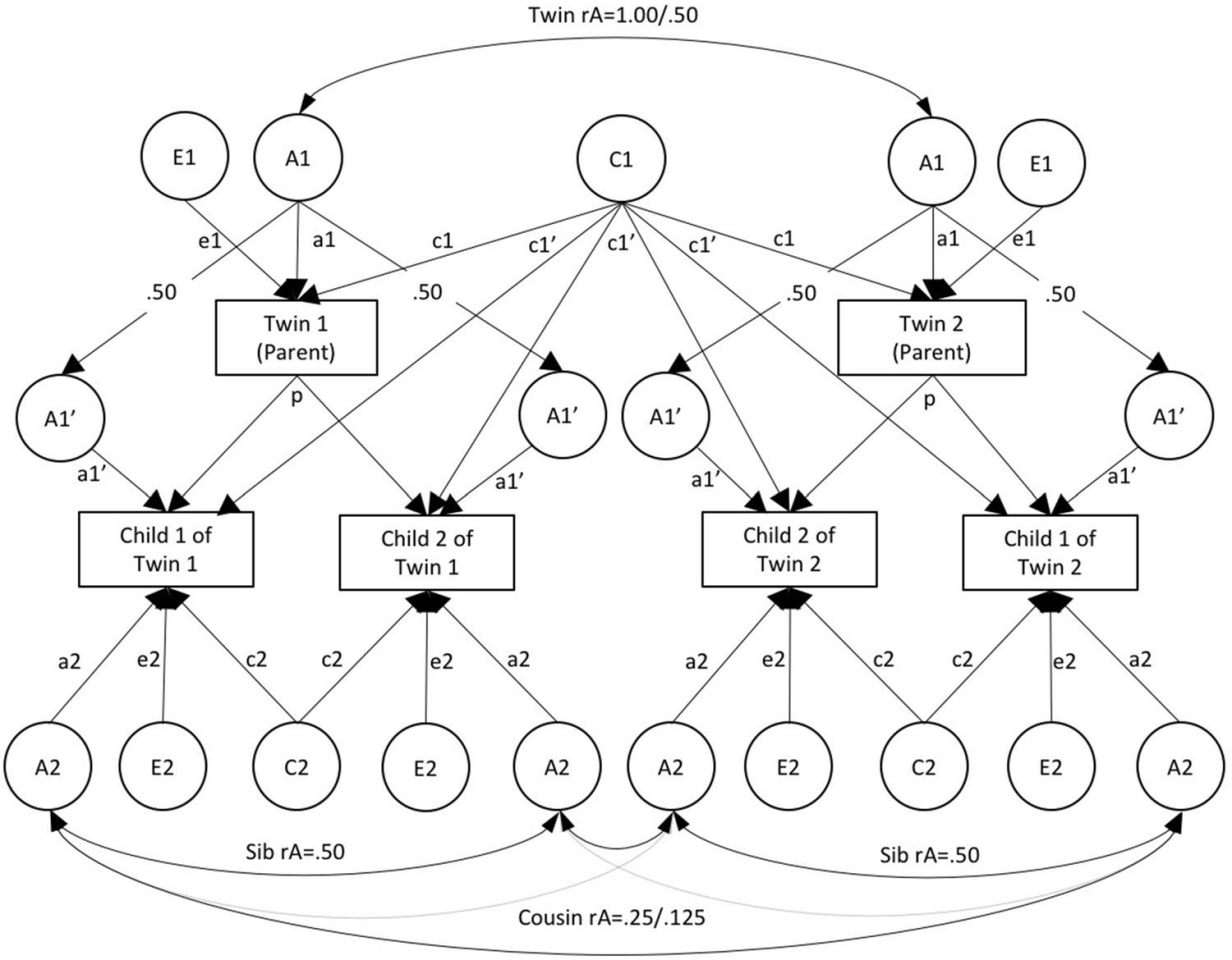
Multiple-children-of-twins structural equation model. Parent phenotype is invariant across offspring (MCoT-inv). *Note*: A1 = additive genetic effects on parental phenotype; C1 = shared-environmental effects on parental phenotype; E1 = nonshared environmental effects on parental phenotype; A1′ = genetic effects common to parental phenotype and offspring phenotype; C1′ = extended family effects whereby the shared environment of the parents influences offspring phjenotype; A2 = familial effects specific to offspring phenotype; C2 = shared-environmental effects on offspring phenotype (not estimable using cousin data); E2 = nonshared environmental effects on offspring phenotype; p = phenotypic effect of parent on offspring; rE = within-parent correlation between E1 for parenting of child 1 and 2. Allows parenting of each child to differ (when necessary this should be allowed to vary according to offspring zygosity). NB the pathway between A1 and A1′ is fixed to 0.50 because parents and children share 50% of their genome. To avoid over complicating path diagrams, variance paths have been omitted, but for all latent factors variance = 1. For A1′ this means that residual variance (after accounting for the path between A1 and A1′) is 0.75

In Fig. 2 we present an extension of the CoT model in Fig. 1b that allows for the inclusion of 2 or more children per parent—the multiple-children-of-twins (MCoT) model.^2^ In this model, the inclusion of siblings in the offspring generation provides sibling covariances with which to estimate the influence of the shared environment on the offspring phenotype (C2). Siblings also share more genetic variance than cousins, increasing power to detect genetic effects in the offspring generation. Including two or more children per parent also provides much more information with which to estimate offspring aetiology: In the MCoT model with two offspring there are two sibling covariances and four cousin covariances per family, compared to one cousin covariance in the standard CoT model. Similarly, the MCoT model with two offspring includes twice as many intergenerational covariances per family (parent-offspring and avuncular) as the CoT model. As such, the MCoT model should have more power to decompose intergenerational associations into a1′, c1′ and p, and to estimate offspring aetiology (see Table 1 for actual power calculations, introduced below). It is worth noting here that in the MCoT model, offspring correlate at 0.25 on the A1′ factor. This is because although both children are equally related to their parent, each child shares a random 50% of their genes with their parent. Importantly, where siblings in the offspring generation are MZ twins they will correlate at 1 on the A1′ factor because they share all of their genes, including the 50% shared with their parent. Such data will need to be modelled accordingly.

The MCoT model presented in Fig. 2 will be useful to researchers interested in associations between parent and offspring phenotypes where the parental phenotype in question is *invariant* across children (e.g., certain diagnoses, or ‘historic’ parental phenotypes such as history of juvenile delinquency). However, many parental phenotypes have the potential to vary between children within the same family (e.g. many parenting phenotypes, psychopathology measured at different time points). As such, the MCoT model in Fig. 2 (hereon referred to as MCoT-inv) must be further extended to deal with parent phenotypes that are *variant* across offspring.

**Fig. 3:**
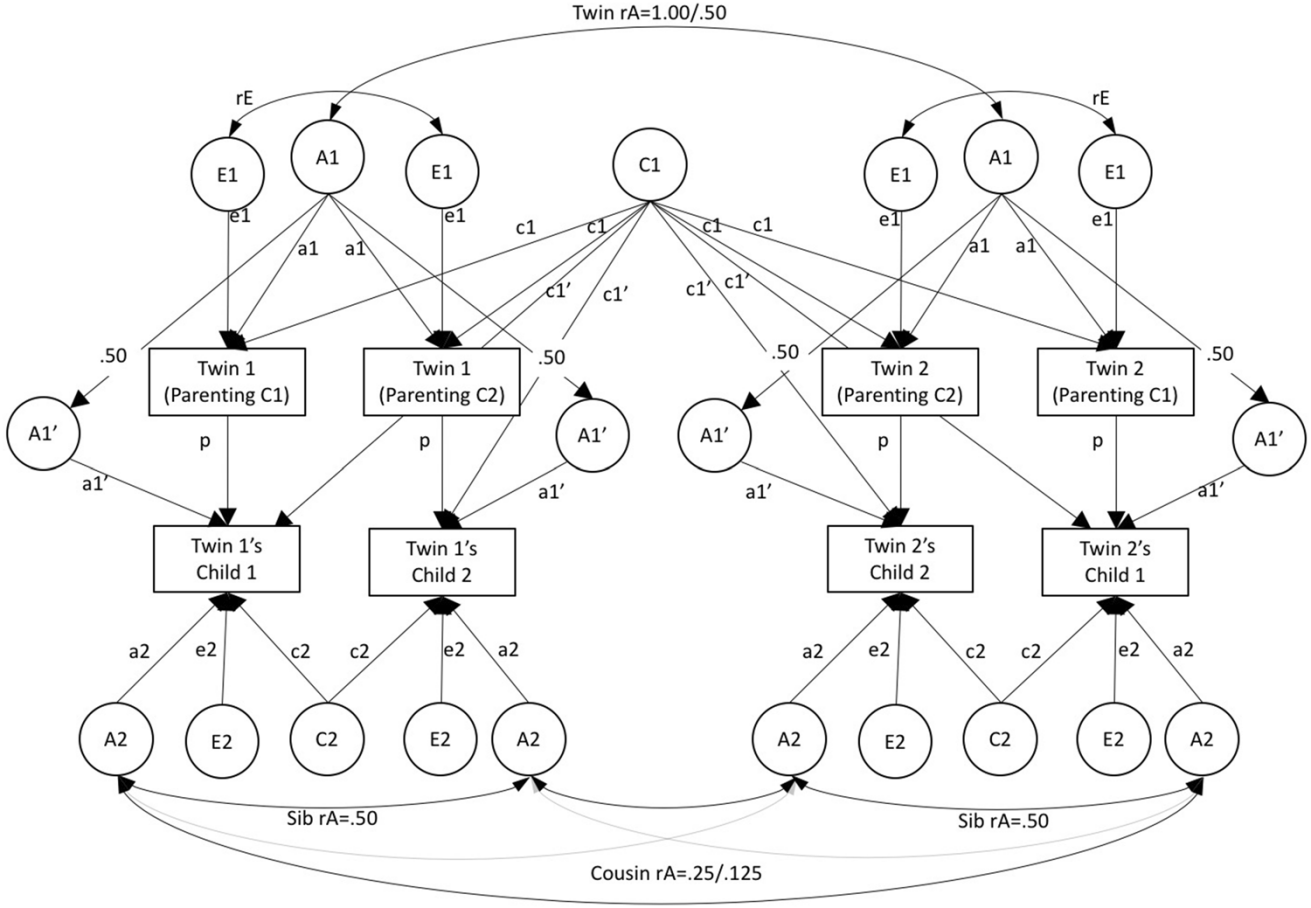
Multiple-children-of-twins structural equation model. Parent phenotype is variant across offspring (MCoT-var). *Note*: A1 = additive genetic effects on parental phenotype; C1 = shared-environmental effects on parental phenotype; E1 = nonshared environmental effects on parental phenotype; A1′ = genetic effects common to parental phenotype and offspring phenotype; C1′ = extended family effects whereby the shared environment of the parents influences offspring phjenotype; A2 = familial effects specific to offspring phenotype; C2 = shared-environmental effects on offspring phenotype (not estimable using cousin data); E2 = nonshared environmental effects on offspring phenotype; p = phenotypic effect of parent on offspring; rE = within-parent correlation between E1 for parenting of child 1 and 2. Allows parenting of each child to differ (when necessary this should be allowed to vary according to offspring zygosity). NB the pathway between A1 and A1′ is fixed to 0.50 because parents and children share 50% of their genome. To avoid over complicating path diagrams, variance paths have been omitted, but for all latent factors variance = 1. For A1′ this means that residual variance (after accounting for the path between A1 and A1′) is 0.75

In extending the MCoT model for use with parent phenotypes that vary between offspring, it is important to consider how to specify the ‘within-person’ correlation that occurs between, for example, Twin 1’s parenting of their first and second child. In some CoT models (Narusyte et al. 2008) such correlations have been specified as A1 + C1, meaning that differences in e.g. the parenting of child 1 and parenting of child 2 are explained by unique environmental influences (E1). This specification is straightforward and makes a certain amount of sense (i.e. it is the same parent). However, it also has the undesirable consequence of constraining within-person correlations to be the same as MZ correlations, and thus brings within-person correlations into the estimation of A, C and E. Another alternative would be to specify the within-person correlation as a Cholesky decomposition, thus freely estimating the association between A, C and E on the first parent phenotype and the second. This may be of use to researchers interested in the effects of parity, e.g. aetiological differences in the parent phenotype for the first vs. second child where data is sorted by birth-order. However, for most situations we advocate a third option, presented in Fig. 3 (MCoT-var). Here, the within-person association is estimated as a combination of A, C, and E, where the correlation between E for e.g. the parenting of child 1 and child 2, is freely estimated (see rE in Fig. 3), thus allowing for differences between the two phenotypes. This does not affect the estimation of A, C or E and does not involve as many assumptions, or require the estimation of as many additional paths, as the Cholesky decomposition. Where the offspring generation comprises multiple zygosities (MZ, DZ, sibs etc.), and where the within-person correlation on the parent phenotype is known to differ by child zygosity (e.g. parenting and any other phenotypes where child-driven evocative rGE is present), a separate rE correlation should be estimated for each zygosity to ensure that estimates are unbiased. Yet another alternative specification would be to include an additional latent factor (N for nuclear family)^3^ to capture the within-person correlation. This would have the benefit of distinguishing between the unique environmental component that makes parents’ behaviour consistent for each of their children (N) vs. the unique environmental component that makes their behaviour different for each child (E1). Such an approach could be useful if the parent phenotype varies between offspring because it was assessed at different time points. However, it would not be appropriate for data in which child zygosity predicts within-person differences in parent phenotype.

While previous studies have not included multiple offspring per twin parent in CoT models, some researchers (Hannigan et al. 2016; Silberg et al. 2010, 2012) have taken another approach to increase their ability to accurately estimate the aetiology of the offspring phenotype, by combining a CoT dataset with data from a children-as-twins (CaT) dataset. In these studies, the CaT dataset comprised data on twins who were of the same age group as the offspring in the CoT dataset, collected using the same instrument. The introduction of the CaT dataset increases power to detect genetic effects in the offspring generation (A2), and allows shared environmental effects to be estimated (C2). A CaT dataset also has the advantage that twins are always the same age as one another, whereas cousins are often not. However, the children-as-twins data inform only on the variance of the child phenotype, they provide *no direct information* on the nature of parent–child covariance. As such, wherever possible we would advise researchers who are considering setting up a CoT/CoS study to collect information on multiple children per parent, as doing so will provide additional sources of covariance with which to decompose intergenerational associations. That said, the use of a CaT dataset does offer obvious advantages in terms of increased power to estimate genetic and environmental effects in the offspring generation, and avoids complications of age difference between siblings/cousins attenuating the estimates if they change with age. It is worth noting here that DZ twinning is heritable, so MCoT studies will generally have the advantage of including twins in both generations.

## Application to simulated data: the power to distinguish routes of intergenerational transmission

The CoT models we present are typically used to assess the extent to which an intergenerational correlation of interest may be attributable to genetic relatedness, and whether an association remains after accounting for relatedness. As discussed, they can also be used to assess the role of the extended family environment, but first we focus on the distinction between genetic vs. phenotypic transmission in the absence of extended family effects. The ability of the model to accurately distinguish phenotypic transmission (i.e. transmission of a social or other nature not involving the passing of genes from parent to child) from genetic transmission is dependent upon the power to detect each of these pathways. For phenotypic transmission, this means the p path. For genetic transmission, this involves genetic effects in the parent generation (a1) and the transmission of these effects to the next generation (a1′). Of all these paths (p, a1, a1′), there is least power to detect a1′. Power to detect the a1′ pathway is dependent upon many factors, including the magnitude of the parent-offspring correlation; the proportion of the correlation that is attributable to genetic overlap; the size of the avuncular relatedness coefficients (0.50 and 0.25 for families in which parents are MZ and DZ twins respectively); the size of the sample; the size of the families within the sample, the MZ:DZ ratio; and the heritability of the phenotypes. In the following power analyses we use simulated data to first demonstrate how the power to detect a1′ is increased by increasing the number of offspring included per parent. We then introduce shared environmental effects into parent and child phenotypes, and explore models including significant extended family effects (c1′) as these are often omitted from CoT models.

In Table 1 we present the results of power analyses of the models presented in Figs. 1, 2 and 3 in scenarios in which intergenerational associations are attributable to genetic and phenotypic effects (models 1-1a, 2-2b, and 3-3b in Table 1); genetic, extended family and phenotypic effects (models 1c, 2c, and 3c); and to extended family and phenotypic effects (models 1d, 2d, and 3d). We present these power analyses with the caveat that in complex SEMs, varying the magnitude of any path can have an impact on the power to detect any other path, so interpretation is not straightforward. We therefore present these analyses simply as an aid to understand how the power to detect the paths of intergenerational transmission (a1′, c1′, p) varies for each of our models. Scripts for running all of the models and power analyses presented in this paper are provided via the supplementary materials. Scripts are designed to simulate data according to given model parameters, so as well as exploring issues of power, readers can explore the covariance structure that might be expected in particular scenarios.

**Table 1:** Results from power analyses exploring power to detect phenotypic (P) genetic (A1′), and family environmental (C1′) intergenerational pathways in CoT, MCoT-inv and MCoT-var models

Model		A1	C1	E1	A2	C2	E2	A1′	C1′	p	Power A1′	Req. N A1′	Power C1′	Req. N C1′	Power p	Req. N p
1	CoT with single child per twin	0.50	0.00	0.50	0.33	–	0.41	0.16	–	0.21	0.82	947 (3788)	–	–	0.98	487 (1948)
1a	CoT with single child per twin. Includes parental C. No C1′	0.50	0.20	0.30	0.33	–	0.41	0.16	–	0.21	0.47	2188 (8752)	–	–	0.79	1029 (4116)
1c	CoT with single child per twin. Includes parental C and C1′	0.50	0.20	0.30	0.33	–	0.39	0.16	0.10	0.07	0.40	2697 (10,788)	0.79	1028 (6168)	0.16	8653 (34,612)
1d	CoT with single child per twin. Includes parental C and C1′. No A1′	0.50	0.20	0.30	0.33	–	0.47	–	0.10	0.21	–	–	0.98	512 (3072)	> 0.99	237 (948)
2	MCoT-inv. Two children per twin. Invariant parent phenotype	0.50	0.00	0.50	0.33	0.00	0.41	0.16	–	0.21	0.97	551 (3306)	–	–	> 0.99	276 (1656)
2a	MCoT-inv. Includes parental C. No C1′	0.50	0.20	0.30	0.33	0.00	0.41	0.16	–	0.21	0.71	1232 (7392)	–	–	0.96	572 (3432)
2b	MCoT-inv with two children per twin. Invariant parent phenotype. Includes child C	0.50	0.00	0.50	0.33	0.20	0.21	0.16	–	0.21	0.93	665 (3990)	–	–	0.98	492 (2952)
2c	MCoT-inv. Includes parental C and C1′	0.50	0.20	0.30	0.33	0.00	0.39	0.16	0.10	0.07	0.64	1452 (8712)	0.94	650 (3900)	0.25	4657 (27,942)
2d	MCoT-inv. Includes parental C and C1′. No A1′	0.50	0.20	0.30	0.33	0.00	0.47	–	0.10	0.21	–	–	> 0.99	253 (1518)	> 0.99	144 (864)
3	MCoT-var with two children per twin. Variant parent phenotype	0.50	0.00	0.50	0.33	0.00	0.41	0.16	–	0.21	0.98	469 (2814)	–	–	> 0.99	237 (1422)
3a	MCoT-var. Includes parental C. No C1′	0.50	0.20	0.30	0.33	0.00	0.41	0.16	–	0.21	0.80	1001 (6006)	–	–	> 0.99	337 (2022)
3b	MCoT-var with two children per twin. Variant parent phenotype. Includes child C	0.50	0.00	0.50	0.33	0.20	0.21	0.16	–	0.21	0.97	512 (3072)	–	–	> 0.99	222 (1332)
3c	MCoT-var with two children per twin. Variant parent phenotype. Includes parental C and C1′	0.50	0.20	0.30	0.33	0.00	0.39	0.16	0.10	0.07	0.72	1218 (7308)	0.94	629 (3774)	0.30	3866 (23,196)
3d	MCoT-var with two children per twin. Variant parent phenotype. Includes parental C and C1′. No A1′	0.50	0.20	0.30	0.33	0.00	0.47	–	0.10	0.21	–	–	> 0.99	242 (1452)	> 0.99	123 (738)

Above we test for the power to detect A1′, C1′ and p pathways using data simulated to fit particular data structures. In each scenario the correlation between parent and child phenotype is 0.35. In models 1-1a, 2-2b, and 3-3b, models are specified such that the parent–child correlation is 40% attributable to genetic relatedness and 60% attributable to phenotypic exposure. In models 1c, 2c, and 3c models are specified such that the parent–child correlation is 40% attributable to the extended family environment and 60% attributable to phenotypic exposure. In models 1d, 2d, and 3d the parent–child correlation is 40% attributable to genetic relatedness, 40% attributable to the extended family environment and 20% attributable to phenotypic exposure. Datasets simulated comprised 1000 complete twin pairs in which each twin had one child in models 1–1d, and two children in all other models. Data is simulated such that 40% of twin pairs are monozygotic. Parent and child phenotypes were normally distributed. All models converged on an accurate solution and all expected variance–covariance matrices mirrored those of the simulated data. As well as giving power in our simulated sample we also give the required N in terms of families (and individuals) necessary to achieve 80% power to detect A1′, p and C1′. NB values given for latent factors (A1, C1, etc.) are variance components, whereas the value given for p is a standardised path coefficient (beta). The effects of A1′, C1′ and p on child phenotype are given as direct effects only and do not include indirect effects e.g. via the path c1′*c1*p. See OpenMx scripts for more details

In all power analyses we simulated data comprising a sample of 1000 twin pairs (40% MZ) with children. We have tried to simulate a scenario that we believe to be representative of a real-world situation—two moderately heritable traits with an intergenerational correlation of 0.35. In the first few power analyses (1-1a, 2-2b, and 3-3b) in Table 1 we specify this association to be 40% attributable to genetic transmission, and 60% attributable to phenotypic transmission. That is, although this association is primarily phenotypic, it would be useful for researchers to be able to identify that over one-third of the association is genetic in origin.

**Fig. 4:**
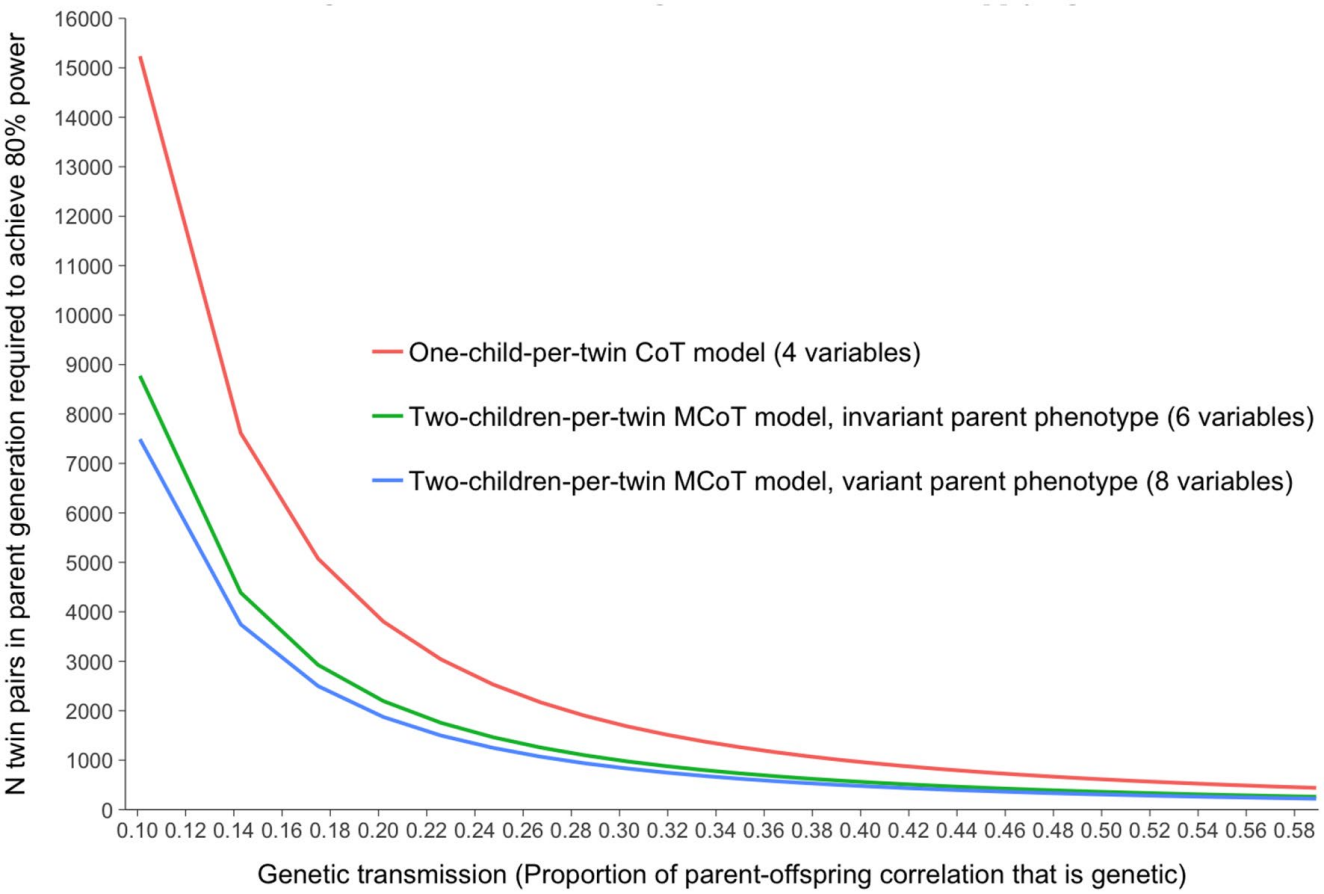
Power to detect genetic transmission using children-of-twin data: Applying three different models to simulated data. *Note*: Data was simulated such that the parent–child phenotypic correlation was always 0.35. Datasets comprised 1000 complete twin pairs where 40% were monozygotic. Only the path a1′ was directly manipulated. Only paths a1′, p and e2 varied. Other specifications were as follows: A1 = 0.50, C1 = 0.00, E1 = 0.50, A2 = 0.33, C2 = 0.00, E2 = residual child variance

In Table 1 it can be seen that power to detect a1′ was lowest in the 4 variable CoT model (model 1a: one-child-per-twin), was greater in the six variable MCoT-inv model (model 2a: two-children-per-twin with invariant parent phenotype), and greater still in the 8 variable MCoT-var model (model 3a: two-children-per-twin with variant parent phenotype). To achieve 80% power to detect a1′ in our example required 947 twin families (twin pairs with children) if using the CoT model, but only 551 when using the MCoT-inv model, and 469 families when using the MCoT-var model. Of course, there are more individuals per family in the MCoT-inv model than the CoT model, but still fewer were required in total (3306 vs. 3788). It is also worth considering that asking twin parents enrolled in a study to provide data on 2+ of their children is likely to be less challenging for researchers than is the recruitment of entirely new twin families. The MCoT-var model comprises just as many individuals as the MCoT-inv model, but includes more data (e.g., additional measurement occasions, or child-specific parenting questionnaire responses). However, the increase in power in the MCoT-var model was such that it required considerably fewer individuals than the MCoT-inv model. In Fig. 4 we illustrate the number of twin pairs required to reach 80% power to detect a1′ of varying magnitude using each of these 3 models. The numbers required decrease as genetic transmission explains an increasing proportion of the correlation between parent and child.

Previously it has been noted that some CoT models have difficulty converging on a correct solution when parental shared environment is non-zero (Narusyte et al. 2008). For this reason we also ran models in which parental shared environment was non-zero (C1 = 0.20: models 1a, 2a, 3a in Table 1). In our simulations these models did converge on a correct solution. However, power to detect a1′ was reduced, with double the number of families required to achieve the same power as simulations where C1 = 0. In simulations where C1 = 0.20 the power was reduced more in the CoT model (1a) than MCoT-inv (2a), than MCoT-var (3a). A non-zero influence of shared environmental effects in the offspring generation (models 2b and 3b) had a far smaller impact upon the ability to detect a1′, demonstrating that this effect is specific to the parental shared environment.

In models 1c, 2c, and 3c we further explored the effect of the shared environment on these models by also including c1′ as a significant contributor to the parent–child association. In these simulations we specified the intergenerational association (rPh = 0.35) to be 40% attributable to genetic transmission, 40% attributable to extended family effects, and 20% attributable to phenotypic transmission. As with all of our analyses, results showed that MCoT-var had more power than MCoT-inv, which had more power than CoT. The inclusion of c1′ further reduced power to detect a1′ in all models. In order to reach 80% power to detect a1′, the CoT, MCoT-inv and MCoT-var models required 2697, 1452, and 1218 families respectively. Power to detect c1′ was far greater than was power to detect a1′, with ~ half or fewer families required to achieve 80% power. Models 1d, 2d, and 3d (in which the intergenerational association was 40% attributable to extended family effects, and 60% attributable to phenotypic transmission) confirm that in the absence of a1′, power to detect c1′ remained very high and considerably higher than a1′ of a similar magnitude.

In the supplementary materials we explore the consequences of not modelling c1′ where it does in fact play a role in intergenerational associations (Table S1). We also explore the consequences of unmodelled dominance effects (Table S2). These analyses are discussed towards the end of this article.

## Application to real data

To further demonstrate the power gained by including multiple children per family in CoT/CoS SEMs, we applied two of the above models to data from the Intergenerational Transmission of Risk (ITOR) project. The ITOR project is based on the Norwegian Mother and Child Cohort Study (MoBa) (Magnus et al. 2016). The MoBa is a prospective population-based pregnancy cohort study conducted by the Norwegian Institute of Public Health. Participants were recruited from all over Norway from 1999 to 2008. The women consented to participation in 41% of the pregnancies. The cohort now includes 114,500 children, 95,200 mothers and 75,200 fathers. The IToR project has linked data from portions of the MoBa to registry pedigrees (i.e. the Norwegian Population Registry and the Medical Birth Registry of Norway), and zygosity information (i.e. based on genotyping, questionnaires, and linkage to the Norwegian Twin Registry). These pedigrees include unique identification numbers for all grandparents, parents, and children that have been listed in the Norwegian Population Registry or Medical Birth Registry.

We examined the association between maternal and offspring height and weight in ITOR. Maternal height and weight prior to pregnancy was self-reported in the 15th week of gestation, and offspring height and weight was obtained via maternal report at age 18 months. The correlation between maternal and child height was 0.28, for weight it was 0.20. First, we fitted a four variable (one child per mother) CoT model to the data. We then fitted MCoT models to the data, allowing for two children per mother. We chose the MCoT-inv model for the analysis of height because maternal height did not differ by offspring. To the weight data we fitted an MCoT-var model, because maternal weight did differ by offspring.

The sample for analysis comprised 10,610 mothers on whom we had data. These included MZ and DZ twin pairs, siblings, and half-siblings (see Supplementary Materials Table S3 for a complete sample breakdown). These individual mothers constituted 4875 complete sibling pairs, and 860 incomplete pairs (i.e. singletons). We had data on 8281 children. In the first analysis (one child per mother), 7090 of these children were included. In the second analysis (up to two children per mother), all were included. In the second analysis there were 1191 complete sibling pairs in the offspring generation. The sibling pairs in the offspring generation also included MZ twin pairs (35), and half-sibling pairs (9).

Parameter estimates from all analyses are displayed in Table 2. For both height and weight, the estimates of parental aetiology were very similar when comparing across the CoT and MCoT models, as should be expected. However, the same was not true for the offspring aetiology, with noticeable differences in parameter estimates and their significance (i.e. confidence intervals).

**Table 2:** Results of of CoT and MCoT analyses of the intergenerational association between maternal height and weight and the height and weight of offspring at age 18 months in the ITOR dataset

Model	A1	C1	E1	A2	C2	E2	A1′	C1′	p	Prop rPh genetic	Prop rPh fam env	Prop rPh phenotypic
Height												
CoT model: one-child per parent	0.86 (0.79, 0.93)	0.04 (0.00, 0.09)	0.10 (0.07, 0.13)	0.87 (0.00, 0.93)	–	0.00 (0.00, 0.80)	0.04 (0.00, 1.00)	0.02 (0.00, 0.17)	0.15 (− 0.24, 0.29)	0.35	0.10	0.56
MCoT-inv model: two-children per parent	0.90 (0.81, 0.93)	0.01 (0.00, 0.07)	0.09 (0.01, 0.12)	0.55 (0.00, 0.72)	0.00 (0.00, 0.26)	0.12 (0.08, 0.20)	0.26 (0.08, 0.72)	0.06 (0.00, 0.18)	0.01 (− 0.16, 0.12)	0.87	0.11	0.02
Weight												
CoT model: one-child per parent	0.68 (0.62, 0.73)	0.00 (0.00, 0.01)	0.32 (0.27, 0.37)	0.34 (0.00, 0.89)	–	0.39 (0.00, 0.85)	0.21 (0.00, 0.28)	0.07 (0.00, 0.11)	0.00 (0.00, 0.13)	0.98	0.02	0.00
MCoT-var model: two-children per parent	0.69 (0.64, 0.74)	0.00 (0.00, 0.02)	0.31 (0.26, 0.36)	0.49 (0.00, 0.81)	0.08 (0.00, 0.31)	0.17 (0.00, 0.57)	0.21 (0.05, 0.27)	0.04 (0.00, 0.08)	0.00 (0.00, 0.09)	0.97	0.03	0.00

A1, C1, E1, A2, C2, E2, A1′, C1′ are given as variance components, p is a path estimate. p^2^ will give the proportion of variance accounted for by p. The final three columns provide the proportion of parent–child covariance accounted for by genetic, extended family environmental, and phenotypic pathways

For height, the results of model fitting (see Table S4 and accompanying text in supplementary materials) revealed that it was possible to drop A1′, C1′ or the p path from the CoT model, indicating that it was not possible to distinguish between the different routes of intergenerational transmission in this analysis. Conversely, in the MCoT model, it was not possible to drop the A1′ path or the C1′ path, indicating that both of these routes were significant. Examination of the MCoT model confirms the importance of genetic factors, with 87% of the mother–child covariance being attributable to genetic transmission. These results would strongly suggest that genetic transmission is the primary reason for correlations between maternal height and the height of their 18-month-old children.

For weight, model fitting results led to the same conclusion for the CoT and MCoT analysis, whereby genetic transmission explained the covariance between maternal pre-pregnancy weight and that of their child at age 18 months. The results of model fitting and confidence intervals highlights the greater power of the MCoT model to detect a1′. While the participation rate of 41% in the MoBa study gives some cause for caution in interpreting these findings, we are not aware of any reason to expect the mechanisms underlying the correlation between mother and child height and weight to vary by participation.

## Discussion

In this article we have demonstrated how to expand CoT models to include multiple children per twin, and how doing so can increase power and solve some of the shortcomings of previous models. Using real data, we have shown that the inclusion of more than one offspring per parent can have a substantive impact on results, even when only a small proportion of the overall sample has more than one child in the study. For the association between mother and child height, the (one child per parent) CoT model could not distinguish genetic from phenotypic transmission. However, once we included complete sib-ships in the offspring generation, our MCoT model clearly demonstrated that this association could best be understood as primarily genetic in nature.

We believe that our extensions to existing CoT models, and findings regarding the associated increases in statistical power, constitute an important step forward and will be informative to researchers as they make use of available CoT and family data. It is also our desire that this article be of use to researchers interested in collecting new data on twins and their children. Our findings emphasise the benefits of collecting data on multiple children per parent. It is also worth considering other ways in which researchers might get the most out of CoT data. For example, the inclusion and retention of childless twins and incomplete pairs in CoT databases is advisable. In typical twin studies (i.e. twins without offspring), the inclusion of incomplete pairs means that twins without a co-twin in the sample can still contribute to the estimation of means and variances, even if they do not contribute information to the estimation of twin covariance and aetiological decomposition. In a CoT study, if only one member of a twin pair has a child, including data on the childless co-twin will also allow this family to contribute to avuncular correlations. Thus, such families can also inform on the nature of intergenerational associations. This is preferable to only including families in which both twins have offspring, and will increase the generalizability of the sample (although of course the inclusion of childless co-twins may not make sense/be possible for all phenotypes).

It is also worth highlighting that the models we present are not only suitable for use with twin data but can easily be adapted for use with population or family databases, where comparisons between MZ and DZ twin families can be accompanied or replaced by comparisons between siblings, half-siblings, cousins, and half-cousins (e.g. as in the ITOR sample in this article). That is, comparisons between any groups of differentially related families can be used in this way. Power will be lower where relatedness coefficients are smaller, but the potential for larger sample sizes will often mitigate this.

Below we attempt to expand and clarify upon the interpretation of the CoT models we have presented, discuss some nuances, and highlight future directions:

### Interpreting CoT models: the phenotypic pathway

The path that we have labelled ‘p’ in Figs. 1, 2, and 3 is intended to capture any association between parent and child phenotype not attributable to genetic relatedness (i.e. not captured by the genetic route of transmission; a1′) or the extended family environment (c1′). We would interpret a significant phenotypic pathway as indicative that one or more of the following is true (conditional on the power considerations discussed earlier in this article): (1) Parental phenotype causally influences the offspring phenotype *above and beyond effects attributable to genetic relatedness and extended family effects*. (2) Offspring phenotype causally influences parent phenotype *above and beyond effects attributable to genetic relatedness and extended family effects*. (3) Parent and offspring phenotype are each associated with a third variable, not included in the model and not captured by shared genetic or extended family effects, that has a causal influence on each, thus creating/inflating their statistical association. Note that these interpretations do not entirely preclude genetic effects from playing a role in parent–child associations—parent and child phenotype can each be under genetic influence, so e.g. the genetically influenced behaviour of a child could have an influence on their parent’s behaviour (and/or vice versa). Such effects would constitute an evocative rGE and in the models presented would load onto the phenotypic pathway. The phenotypic pathway suggests that there may be a role for *exposure—*that is, after accounting for genetic relatedness, the parent phenotype may influence child phenotype, and/or vice versa. The p path has previously been described using several different terms including ‘non-genetic’, ‘environmental’, and ‘social’. We have chosen to use the term ‘phenotypic’, as this carries fewer implications about the origin of the association.

In terms of the way that the SEMs in Figs. 1, 2 and 3 are specified, the phenotypic ‘p’ pathway is a causal path. That is, it is a single-headed arrow running from parent to child. However, it is important to note that no assumptions should be made regarding the direction of causation unless the data permits (e.g. by temporal precedence). Previously, these models have been used on cross-sectional data, so any association should be interpreted as correlational—not necessarily indicative of a causal influence of parent on child (or indeed vice versa). The path is modelled as causal (i.e. a single headed arrow) because it is not possible in SEM to model a path between two endogenous variables as correlational (i.e. a double headed arrow). It is however possible to re-specify these models such that the direction of causation runs from child to parent. Doing so will result in the same conclusions as using the specifications and models we present here.

### Interpreting CoT models: the A1′ pathway

The A1′ pathway in the CoT models presented captures covariance between the parent and child phenotypes that is attributable to their genetic relatedness. That is, this pathway is intended to estimate intergenerational genetic transmission. It is worth noting here that in recent years several papers have included reports of non-significant A1′ pathways where some genetic transmission might have been expected. For example, if depression is heritable, why is none of the intergenerational association between parent and child depression attributable to genetic transmission (McAdams et al. 2015; Silberg et al. 2010; Singh et al. 2011)? There are several reasons that CoT models may find no genetic associations where they are expected: First, the models may be underpowered. The identification of intergenerational genetic transmission in a CoT model relies upon differences between MZ avuncular correlations, and DZ avuncular correlations. The genetic relatedness coefficients in these relationships are 0.50, and 0.25 respectively. This means that statistical power is lower than, for example, when conducting multivariate genetic analyses within a generation of twins, in which correlations are decomposed using differences between MZ and DZ correlations (where relatedness coefficients are 1.00 and 0.50 respectively). We have demonstrated in this article that this is a reason to include multiple children per parent wherever possible.

Second, there is perhaps less reason to expect the intergenerational associations examined to be attributable to genetic relatedness than may commonly be assumed. For example, when studying the intergenerational transmission of psychopathology, datasets often comprise adult twins with child/adolescent offspring. Given that genetic innovation is a common finding in the literature (i.e. the genetic architecture of traits changes across the lifespan) (e.g. Hannigan et al. 2017), it is perhaps not surprising that genetic factors involved in psychopathology in adults do not always correlate perfectly with those involved in child or adolescent psychopathology. It is also possible that cohort effects play a role—i.e. genetic factors involved in psychopathology in one generation may not be the same as those in subsequent generations. It is known that the genetic architecture of a trait can be dependent upon the environment in which it is expressed (Rutter et al. 2006). So, given the differences in environmental experiences of e.g. somebody who grew up in the 1960s and their offspring who grew up in the 1990s, it should perhaps not be too surprising if we find that genetic overlap between parent and child is not perfect, even if we measure ostensibly the same phenotype in each generation.

Previously, when presenting findings from CoT studies we have encountered questions from some reviewers and researchers along the lines of “if children-as-twin studies report that the association between, for example, parenting and behavioural problems is partially genetic (e.g. McAdams et al. 2013), then why is the A1′ pathway often non-significant in CoT models assessing similar associations?” This is a common query based on a common misunderstanding, so we thought it worthwhile outlining a response. It is important here to consider the differences in information included in CaT vs. CoT studies. In a CaT sample, if a parenting measure is found to be heritable, then this indicates that *child genes* influence/correlate with the parenting that they receive. Often, in these studies, child genes associated with the parenting they receive are also associated with child behaviour (McAdams et al. 2013). In a CoT sample, if a parenting phenotype is heritable, then this indicates that *parent genes* influence parenting. In the CoT models presented in the preceding Figures, where offspring phenotype is heritable, this indicates that child genes influence offspring phenotype. So in CaT models, genetic correlations between parenting and child behaviour indicate that children’s genes influence both. In the above CoT models, genetic correlations would demonstrate an association between parent genes involved in parenting, and child genes involved in child phenotype. As such, these models are not directly comparable and tell us different things about the nature of parent–child associations. It is also worth noting here that in CaT studies, the aetiology of parenting variables can be greatly affected by the reporter, with parents reporting that they treat their children very equally (resulting in large C estimates), and children reporting otherwise (resulting in far smaller C estimates). In CaT studies the heritability of parenting is calculated based on how similarly individual parents parent their MZ vs. DZ twin children. In contrast, CoT studies involve comparing how similar MZ vs. DZ twins are in parenting their own children. As such, estimates of the heritability of parenting derived from CoT samples are less susceptible to reporter effects.

### The shared environment in CoT models

The shared environment is typically described as “environmental effects that make members of a family similar to one another” or “environmental effects that make siblings similar to one another”. Such definitions are straightforward when working with standard twin datasets, but less so with CoT and CoS datasets, where several sib-ships can be nested within nuclear families, which in turn are nested within extended families. It is possible with extended family data to specify several types of shared environment. In the above models we have included C1—environmental effects that makes siblings in the parent generation similar to one another, and C2—environmental effects that make siblings in the offspring generation similar to one another (besides those going via the p path). We have also included a c1′ path, via which extended family effects can be transmitted that can explain some or all of the correlation between parent and child phenotypes. Other possible shared environmental effects are possible, and several have been modelled in Swedish population registry data using intergenerational models similar to those we present here (Chang et al. 2014; Kuja-Halkola et al. 2014; Latvala et al. 2015).

While working with simulated data, we found that when parental shared environment (C1) was non-zero, power to detect A1′ reduced substantially, even when the shared environment played no role in explaining the intergenerational association. This finding aligns with reports by Narusyte et al. (2008), that their bidirectional model had problems converging on the correct solution when parental shared environment was present. Nonetheless, as highlighted by Narusyte et al. (2008), this is unlikely to be a major concern for most researchers using CoT/CoS data, as a majority of parental phenotypes likely to be included in these models (parenting, personality, psychopathology) are not under the influence of shared environmental influences in adulthood. That said, it is of course possible to think of exceptions to this rule (e.g. education, SES).

We also explored models designed for those situations in which the shared environment may play a role in explaining intergenerational associations via the pathway c1′. Where genetic and extended family environment effects explained equal portions of the intergenerational association, power to detect c1′ was higher than power to detect a1′. The presence of c1′ also led to a slight reduction in power to detect a1′ relative to models in which c1′ was held at zero. Importantly, failing to allow for a possible role of c1′ when it did in fact play a role in explaining intergenerational associations led to an overestimation of a1′ and underestimation of p. This is an important finding given that many CoT models to date have not allowed for the possibility of an effect of the extended family environment. To ensure accurate results we would encourage researchers to include c1′ paths (or similar) in their models. If clearly non-significant then these paths can be dropped from models in order to maximise power to detect a1′.

#### Dominance

In the models presented in Figs. 1, 2 and 3 we have not included latent factors to account for the potential effects of non-additive genetic effects, or *genetic dominance*. In twin models, latent factors can be added to capture effects attributable to interactions between alleles at a locus (e.g. dominance; D). Such interactions increase the similarity between siblings whenever they share the same alleles at a given locus. That is, when siblings inherit the same genes as one another from both their mother and their father. MZ twins are genetically identical, so they share all dominance genetic effects with one another. Full siblings inherit the same genes from both their mother and their father at a given locus 25% of the time on average. Thus, for MZ twins the correlation for D (rD) is 1.00, whereas for DZ twins and full siblings rD = 0.25. For other family dyads (cousins, half-siblings, parent–child etc.) rD = 0.00 because they do not share both parents. In this paper we have focussed on the use of CoT/CoS data in understanding the nature of intergenerational associations. We have therefore omitted dominance effects from our models because such effects are not directly transmitted from one generation to the next. This is because children only inherit one copy of each gene from each parent. It is also generally not possible using twin data to estimate the effects of dominance and the shared environment at the same time. However, in the scripts provided in the supplementary materials we do allow for parent (D1) and child (D2) dominance effects for use with phenotypes in which dominance is present. In simulations included in the supplementary materials we explored the consequences of omitting significant parental dominance effects from our models. Generally, the models we present appear to deal with unmodelled dominance well, and conclusions regarding the nature of intergenerational associations should not be biased except for instances in which the extended family environment also plays a role (see Supplementary Materials and Table S2 for more details).

### Limitations and considerations

As with all models, CoT and MCoT models have limitations that should be taken into consideration. Many of these limitations have been discussed elsewhere. For example, a CoT-specific version of the equal environments assumption (EEA) is that the children of MZ twins are not exposed to their parent’s co-twin any more than are the children of DZ twins. Although, unlike the EEA in standard twin studies, in CoT studies it is quite easy to account for this by measuring the amount of avuncular contact and including in the model (Koenig et al. 2010). Eaves et al. (2005) have discussed the “dyadic variables issue”, whereby CoT models may not work when parental phenotypes are the product of the interaction between people (e.g. divorce) rather than the characteristic of a single parent.

Other considerations involve the fact that age differences between cousins and siblings in the offspring generation could lead to biases in estimates of offspring aetiology where age is associated with aetiology. To counter this, studies can be designed so that data is collected only when offspring reach a particular age. Alternatively, constraints can be applied to data included in models to minimise age differences (e.g. only including relatives within 5 years of each other). It would also be possible to regress the effects of age out of phenotypes before entering into SEMs, or to include age as a covariate. While we have not explored the possibility of modelling sex differences in our models, it would be possible to do so, although we would anticipate that power considerations would mean that very large samples are required to accurately identify any differences.

### Alternative methods

In the present article we have followed others (e.g. D’Onofrio et al. 2003; Keller et al. 2009; Narusyte et al. 2008, 2011; Silberg and Eaves 2004; Silberg et al. 2010, 2012) in adapting well-established biometric SEMs that have previously been applied to extended twin family data. We are hopeful that by adapting this already well-established approach to intergenerational analyses, our models will prove useful to the field. For example, previously researchers have demonstrated how to add spouses to these models to account for the effects of assortative mating (Keller et al. 2009; Silberg and Eaves 2004; Silberg et al. 2010, 2012) so it would be possible to include spouses in the models that we have presented. However, as is always the case with complex data, it is possible to model CoT/CoS data in many different ways. Kuja-Halkola et al. (2014) have previously applied biometric SEMs to intergenerational CoS data extracted from Swedish population registries (see also Chang et al. 2014; Latvala et al. 2015). The models that they have used are specified differently to those we have presented in this article, in that instead of estimating a1′, c1′ and p, they estimate correlations between parent A and child A, parent C and child C (additional shared environmental factors are often estimated as well), and parent E and child E. They interpret a significant E correlation as indicating that an association between parent and child phenotypes remains after accounting for shared genetic and familial environmental effects. We don’t see any reason why the conclusions regarding the nature of intergenerational associations should differ dependent upon whether researchers use the models presented by Kuja-Halkola and colleagues or those we present here.

While the focus of this article has been on SEM techniques, there are of course alternative approaches that can be used with MCoT/CoS data, including a range of multilevel regression models with modelled genetic relatedness. Previously such approaches have been taken with CoT/CoS data to explore whether associations between parent and child persist after accounting for familial confounding (D’Onofrio et al. 2005; Jundong et al. 2012; Slutske et al. 2008). It may also be possible to decompose covariance into genetic and environmental components using such methods (i.e. Rabe-Hesketh et al. 2008), although to our knowledge this has not been done with CoT data. Another possible way to model multiple children per twin in CoT data would be to use multi-level SEM techniques (Rabe-Hesketh et al. 2007). Such techniques combine the advantages of SEM (ability to specify/identify latent variables, and to graphically represent and explore complex relationships between many variables) with those of multilevel modelling (ability to deal with complex data structures, and explore relationships at different levels within a dataset). To our knowledge, such models have yet to be developed or applied to CoT/CoS data, but it is possible that this will happen in the future.

### Future directions

Because our focus for this paper was on the benefits of including multiple offspring in CoT models, we did not include spouses in the models that we have presented. However, it is possible to incorporate spousal information. For example, several extended twin-family models include the spouses of twins (Keller et al. 2009), and spouses have been included in CoT models (Silberg and Eaves 2004; Silberg et al. 2010, 2012). In many instances, it may be of importance to incorporate spouses into intergenerational models, as aetiological estimates may be biased by assortative mating, and it is known that assortative mating is common across physical, behavioural, and cognitive phenotypes (Nordsletten et al. 2016; Plomin and Deary 2015; Stulp et al. 2017). In the nuclear twin family model and stealth model, twin-spouse correlations are modelled as ‘primary assortment’, meaning that twins choose spouses who are phenotypically similar to them. However, alternative explanations for twin-spouse similarity exist, including social homogamy, whereby partners are chosen based on sociocultural similarity; and phenotypic convergence, whereby partners become more alike over time. Assortative mating is a complex issue, the implications of which have yet to be fully explored (Heath et al. 2014; Plomin et al. 2016). In the future, it will be important to consider the impact of assortative mating on the parameter estimates obtained using CoT models.

Besides incorporating assortative mating into CoT models, future model developments should focus on the creation of multivariate CoT models. At present, CoT models are focussed on the decomposition of associations between one parent phenotype and one child phenotype. However, for many research questions, it would be informative to include additional phenotypes. For example, when studying associations between parent and child psychopathology, it would be of interest to incorporate parenting behaviours or relationship measures that are hypothesised to mediate or moderate associations. It is also known that many parental mental health phenotypes are predictive of multiple outcomes in offspring. For example, parental depression is associated with a host of negative outcomes in children (Natsuaki et al. 2014). Likewise, most child outcomes are associated with multiple predictors in the parent generation. Being able to simultaneously model such associations would be beneficial and may aid attempts to identify key intervention targets with which to maximise impact. While it is possible to assess the direction-of-effects between parent and child phenotypes using cross-sectional CoT data (Narusyte et al. 2008), being able to make use of longitudinal CoT data should also be a priority for future model development.

In summary we have demonstrated that by incorporating multiple offspring per parent, the power of CoT and children-of-sibling models can be increased. Improving such models will aid in future efforts to disentangle potential social influences from genetic transmission in the study of intergenerational effects. Understanding the nature of intergenerational transmission has the potential to inform both on our understanding of genetics and on the influence that parents and children have on one another. For example, where researchers establish that parent and offspring phenotypes are linked genetically, then we learn something about the genetic factors involved: They persist into adulthood (if offspring are children), they are pleiotropic (if the phenotypes are distinct), they persist across time (i.e. they associate with the same phenotype in cohorts born in different eras), and they explain a portion of familial similarity. Where well-powered studies indicate that genetic transmission does not play a role, then it will be important to consider what processes might explain associations. And while it is not the case that associations under genetic influence are not amenable to intervention, when effects do remain after accounting for relatedness, it will be possible to guide intervention efforts to focus on those familial risk factors that constitute true environmental influences on child development.

## Electronic supplementary material

Below is the link to the electronic supplementary material.

Supplementary material 1 (PDF 73 KB)

Supplementary material 2 (R 61 KB)

## Footnotes

1 For a detailed introduction to the use of children-of-twins data, the analytical methods frequently applied to such data, and a review of the empirical literature we refer readers to McAdams et al. (2014).

2 In the figures and examples we provide, our MCoT models include two children per parent. However, it is perfectly possible to include three or more offspring per parent by simply extending the accompanying matrices (see OpenMx scripts for more details).

3 Credit goes to an anonymous reviewer for this suggestion.
