# Supplementary material for: Revisiting the Children-of-Twins Design: Improving Existing Models for the Exploration of Intergenerational Associations
Source: Behav Genet. 2018 Jun 30;48(5):397–412. doi: 10.1007/s10519-018-9912-4 (PMC6097723; doi:10.1007/s10519-018-9912-4)
Supplement: Supplementary file 1 — Supplementary material 1 (PDF 73 KB) [file 10519_2018_9912_MOESM1_ESM.pdf]

# Supplementary materials

The extended family environment

To explore the consequences of not modelling c1' where c1' does play a role in explaining the intergenerational association, we simulated 3 sets of data as specified in table S1. In each example, c1' explains varying portions of the intergenerational association (where rPh=.35). We fitted CoT and MCoT models to these datasets in which c1' was not included in the model (i.e. it was fixed to zero). This is an important simulation because c1' has often been omitted from the models fitted to CoT data in the literature. In each example, and with each model (CoT, MCoT-inv and MCoT-var), the omission of c1' resulted in an inflated A1' estimate and a reduced p estimate and E2 estimate, the magnitude of this effect being dependent upon the size of the omitted c1' influence. Previously reported findings within the literature should be interpreted with this in mind.

Table S1. Exploring the consequences of not modelling intergenerational transmission via the extended family environment (C1')

| | A1 | C1 | E1 | A2 | C2 | E2 | A1' | C1' | p | %A1' | %C1' | %P |
|---|---|---|---|---|---|---|---|---|---|---|---|---|
| **1. Data simulated:** | **.50** | **.20** | **.30** | **.33** | **.00** | **.39** | **.16** | **.10** | **.07** | **40%** | **40%** | **20%** |
| CoT estimates | .56 | .14 | .30 | .27 | - | .00 | .71 | - | .03 | 91% | - | 9% |
| MCoT-inv estimates | .58 | .12 | .30 | .30 | .00 | .00 | .68 | - | .03 | 92% | - | 8% |
| MCoT-var estimates | .58 | .12 | .30 | .30 | .00 | .01 | .66 | - | .03 | 90% | - | 10% |
| **2. Data simulated:** | **.50** | **.07** | **.43** | **.33** | **.00** | **.39** | **.16** | **.07** | **.14** | **40%** | **20%** | **40%** |
| CoT estimates | .52 | .05 | .43 | .43 | - | .09 | .42 | - | .11 | 68% | - | 32% |
| MCoT-inv estimates | .50 | .06 | .44 | .34 | .00 | .14 | .46 | | .11 | 69% | | 30% |
| MCoT-var estimates | .50 | .06 | .44 | .35 | .00 | .15 | .44 | - | .11 | 68% | - | 32% |
| **3. Data simulated:** | **.50** | **.04** | **.46** | **.33** | **.00** | **.36** | **.195** | **.03** | **.155** | **45%** | **10%** | **45%** |
| CoT estimates | .51 | .03 | .46 | .36 | - | .23 | .33 | - | .14 | 59% | - | 41% |
| MCoT-inv estimates | .51 | .03 | .46 | .34 | .00 | .25 | .33 | - | .14 | 59% | - | 41% |
| MCoT-var estimates | .51 | .03 | .46 | .34 | .00 | .25 | .33 | - | .14 | 59% | - | 41% |

In each simulation, the parent-child correlation was r=.35. A1, C1, E1, A2, C2, E2, A1', C1' are given as variance components, p is a path estimate. $p^2$ will give the proportion of variance accounted for by p. The final 3 columns provide the proportion of parent-child covariance accounted for by genetic, extended family environmental, and phenotypic pathways.

Dominance

In biometric models the effects of genetic dominance can be captured via the inclusion of latent dominance (D) factors. These can be used to capture effects attributable to interactions between alleles at a locus. Such interactions increase the similarity between siblings whenever they share the same alleles at a given locus. That is, when siblings inherit the same genes from both their mother and their father. For MZ twins this is always the case and for full siblings this happens 25% of the time on average. Thus for MZ twins the correlation for D (rD) is 1.00, whereas for DZ twins and full siblings rD=.25. For other family dyads (cousins, half-siblings etc.) rD=0.00 because it is necessary to share both parents in order for rD>.00. Dominance effects are not directly transmitted from one generation to the next.

Because it is not usually possible to estimate the effects of dominance and the shared environment at the same time using a twin sample (unless e.g. pairs of half-sibs and cousins are also included), researchers have to choose between estimating dominance or estimating shared environmental effects. We therefore thought that it would be of interest to run some simulations in which both C1 and D1 play a role in explaining parental phenotype, to explore the impact of dominance effects on the estimates derived from our models when dominance is not explicitly modelled. We simulated 2 datasets in which both C1 and D1 are significant (Table S2). In the first, the parent-child correlation was 40% attributable to genetic relatedness and 60% attributable to phenotypic exposure. In this scenario, in our fitted models, A1 was inflated and C1 was deflated. Because A1 was inflated, A1' reduced in magnitude. Thus, the proportion of the intergenerational association explained by genetic transmission remained the same.

In the second scenario, the extended family environment also played a role in explaining the parent-child correlation, such that the association was 40% attributable to genetic relatedness, 20% attributable to the extended family environment and 40% attributable to phenotypic exposure. In this scenario, the effects on each parameter were similar to the first scenario. However, because the presence of unmodelled dominance deflated the C1 estimate, the apparent roles of the extended family environment and phenotypic exposure in explaining the intergenerational association were reduced, with the role of genetic effects being increased. The CoT, MCoT-inv and MCoT-var all performed similarly in these simulations.

Table S2. Exploring the consequences of not modelling genetic dominance (D1) in CoT models

| | A1 | C1 | D1 | E1 | A2 | C2 | D2 | E2 | A1’ | C1’ | p | %A1’ | %C1’ | %P |
|---|---|---|---|---|---|---|---|---|---|---|---|---|---|---|
| **1. Data simulated** | **.50** | **.15** | **.15** | **.2** | **.33** | **.00** | **.00** | **.41** | **.16** | **.00** | **.21** | **40%** | **0%** | **60%** |
| CoT estimates | .73 | .07 | - | .20 | .38 | - | - | .41 | .11 | - | .21 | 40% | - | 60% |
| MCoT-inv estimates | .72 | .08 | - | .20 | .36 | - | - | .43 | .11 | - | .21 | 41% | - | 59% |
| MCoT-var estimates | .72 | .08 | - | .20 | .36 | - | - | .43 | .11 | - | .21 | 41% | - | 59% |
| **2. Data simulated** | **.50** | **.07** | **.15** | **.27** | **.33** | **.00** | **.00** | **.39** | **.16** | **.07** | **.14** | **40%** | **20%** | **40%** |
| CoT estimates | .70 | .03 | - | .28 | .25 | .08 | - | .41 | .18 | .08 | .12 | 51% | 14% | 35% |
| MCoT-inv estimates | .69 | .04 | - | .28 | .29 | .01 | - | .37 | .17 | .08 | .12 | 49% | 15% | 36% |
| MCoT-var estimates | .68 | .04 | - | .28 | .29 | .01 | - | .38 | .16 | .08 | .13 | 48% | 15% | 36% |

In each simulation, the parent-child correlation is r=.35. A1, C1, E1, A2, C2, E2, A1’, C1’ are given as variance components, p is a path estimate. $p^2$ will give the proportion of variance accounted for by p. The final 3 columns provide the proportion of parent-child covariance accounted for by genetic, extended family environmental, and phenotypic pathways.

Table S3. Composition of the ITOR sample used to assess associations between maternal and child height and weight

| Parental sib-ship | N complete pairs in adult generation (incomplete pairs) | Total N children | N children in analysis using ≤1 child per parent | N complete sibling pairs in offspring generation |
|---|---|---|---|---|
| MZ twins | 87 (1) | 136 | 114 | 22 |
| DZ twins | 48 (4) | 76 | 65 | 11 |
| Siblings | 4425 (780) | 7611 | 6508 | 1103 |
| Paternal half siblings | 186 (39) | 268 | 241 | 27 |
| Maternal half siblings | 129 (36) | 190 | 162 | 28 |

Table S4. Results of model fitting for the association between maternal and child height and weight in the ITOR dataset

| | -2LL (df) | AIC | $\chi^2$ (Δdf) | *p* |
|---|---|---|---|---|
| Height | | | | |
| 1. CoT model | | | | |
| Full model | 101290.96 (17690) | 65910.96 | | |
| A1'=0 | 101291.39 (17691) | 65909.39 | 0.43 (1) | .51 |
| C1'=0 | 101292.00 (17691) | 65910.02 | 1.07 (1) | .30 |
| p=0 | 101292.10 (17691) | 65910.13 | 1.17 (1) | .28 |
| 2. MCoT-inv model | | | | |
| Full model | 107030.51 (18880) | 69270.51 | | |
| A1'=0 | 107039.10 (18881) | 69277.08 | 8.56 (1) | <.01 |
| C1'=0 | 107034.70 (18881) | 69272.73 | 4.22 (1) | .04 |
| p=0 | 107030.50 (18881) | 69268.50 | 0.00 (1) | 1.00 |
| Weight | | | | |
| 1. CoT model | | | | |
| Full model | 194458.15 (16975) | 160508.15 | | |
| A1'=0 | 194462.26 (16976) | 160510.26 | 4.11 (1) | .04 |
| C1'=0 | 194458.51 (16976) | 160506.51 | 0.36(1) | .55 |
| p=0 | 194458.15 (16976) | 160506.15 | 0.00 (1) | 1.00 |
| 2. MCoT-var model | | | | |
| Full model | 233898.55 (20729) | 192440.55 | | |
| A1'=0 | 233907.6 (20730) | 192447.6 | 9.05 (1) | <.01 |
| C1'=0 | 233898.8 (20730) | 192438.8 | 0.29 (1) | .59 |
| p=0 | 233898.55 (20730) | 192438.5 | .00 (1) | 1.00 |

Model fitting notes: In the CoT model for height, E2 was estimated as .00. Such a low non-shared environment estimate is unusual, as child measurement error should load on to E2, so E2 is expected to be non-zero. In the MCoT-inv model for height, E2 was estimated as .11 and significant. While we haven't encountered zero E2 estimates in these models before, we felt that this warranted further

investigation. Additional analyses revealed that in the MCoT-inv model it was only the inclusion of MZ pairs in the offspring generation that ensured that E2 was estimated as >.00. When these MZ pairs were excluded, E2 was estimated at .00 again. We believe that this estimation of a very low E2 arose due to our use of sibling and cousin data to calculate the aetiology of a highly heritable trait with a genuinely low measurement error, combined with high sibling and cousin correlations in the data. In the model specification, the only difference between sibling covariance and child variance is E2 and the scaling multipliers on A2 and A1' (see below equations and OpenMx scripts). Because sibling and cousin covariances were high, there was no variance left for E2 to explain once A2 and A1' were scaled up. The introduction of MZ offspring introduced a ceiling for child covariances that was below child variance, thus granting the model the ability to estimate E2 as the difference between child variance and MZ covariance.

$$
\begin{aligned}
\text{Offspring variance} &= A2 + C2 + A1' + P*V1 + 2(.5*a1'*a1*p) + C1' + 2(p*c1*c1') + E2 \\
\text{Sibling covariance} &= .5*A2 + C2 + .25*A1' + P*V1 + 2(.5*a1'*a1*p) + C1' + 2(p*c1*c1') \\
\text{Cousin covariance} &= .125*A2 + C2 + .125*A1' + P*((.5*A1)+C1) + 2(.25*a1'*a1*p) + C1' + 2(p*c1*c1') \\
\text{MZ covariance} &= A2 + C2 + A1' + P*V1 + 2(.5*a1'*a1*p) + C1' + 2(p*c1*c1')
\end{aligned}
$$
